# Sustainable leadership and wellbeing of healthcare personnel: A sequential mediation model of procedural knowledge and compassion

**DOI:** 10.3389/fpsyg.2022.1039456

**Published:** 2023-01-17

**Authors:** Ghulam Abid, Francoise Contreras, Susanne Rank, Sehrish Ilyas

**Affiliations:** ^1^Kinnaird College for Women University, Lahore, Pakistan; ^2^School of Management and Business, Universidad del Rosario, Bogota, Colombia; ^3^University of Applied Sciences Mainz, Mainz, Rhineland-Palatinate, Germany; ^4^Lahore College for Women University, Lahore, Punjab, Pakistan

**Keywords:** sustainable leadership, procedural knowledge, compassion, wellbeing, healthcare settings

## Abstract

**Introduction:**

In healthcare organizations, saving patients’ lives while maintaining the staff’s wellbeing, performance and competencies were challenging during the COVID-19 pandemic. Although the complexity of healthcare settings is widely recognized, the pandemic evidenced the necessity of attending to the employees’ wellbeing in such a sector. This research aims to examine the effect of sustainable leadership on wellbeing of healthcare personnel. Furthermore, we also evaluate whether procedural knowledge and compassion act as mediators in such a relationship.

**Methods:**

The hypothesized model was tested in healthcare organizations in a South Asian country, and the data were collected during the pandemic crisis. A total of 366 health personnel (physicians and nurses) participated in this research. With Hayes’ PROCESS macro, we examined all the direct and indirect paths, including sequential mediation.

**Results:**

The findings confirm the impact of sustainable leadership on wellbeing and this relationship is also mediated by procedural knowledge and compassion.

**Discussion/conclusion:**

Sustainable leadership fosters wellbeing among healthcare workers *via* the sequential mediation of procedural knowledge and compassion. Study findings suggest that sustainable leaders can trigger procedural knowledge among employees which in turn crafts the state of compassion in them that leads to their wellbeing. Theoretical and practical implications are discussed in light of study findings.

## 1. Introduction

Common goodness “with a higher purpose for our society and planet” is the logic behind the sustainability (Contreras and Abid, 2022), which is formulated within the sustainable developmental goals (SDG) of United Nations (United Nations, 2015). Coping with the grand challenges of the 21st century (Aust et al., 2020; Elahi et al., 2022), like global climate change, pandemics and inequality of wealth, sustainable strategies and leadership (Hallinger and Suriyankietkaew, 2018) are currently required for leading every organization to achieve sustainable performance (Iqbal et al., 2020a; Javed et al., 2021). For example, the heat waves that impact the world because of the temperature increase are affecting mainly those densely populated regions already-hot places (Xu et al., 2020) forecast the critical need to have a healthcare infrastructure to attend to their necessities (Bavel et al., 2020). In addition, the pandemics like the current Coronavirus might occur more frequently in the near future as forecasted by EMRO WHO (2022); it is a matter of “when,” not “if” which will impact our society as a whole (Iftekhar et al., 2021).

Consequently, hospitals’ staff not only had to work at the limit during the COVID-19 pandemic—that still persists—but might continue in the emergency status in the near future. This situation shows the need for scholars to focus their attention on the wellbeing of health personnel as a priority issue to address (Qaiser and Abid, 2022; Ilyas et al., 2022a, 2022b). In the healthcare setting, employees’ wellbeing acquired special relevance due to its relationship with performance.

During the heavy burden due of the pandemic, the primary purpose of the caring profession and healthcare organizations was to save patients’ life by continuously enhancing their wellbeing (De Kock et al., 2021; Ilyas et al., 2022a, 2022b). However, not only do the patients require attention, but the health staff also needs it. The COVID-19 situation in hospitals heavily impacted the wellbeing of the staff, especially in times of uncertainty and urgency in the highly stressful work environment (Petrella et al., 2021; Sun et al., 2021). Digby et al. (2021) recently evaluated hospital employees in Australia and identified a high level of anticipatory anxiety due to altered working conditions, isolation, and uncertainty caused by the pandemic. There is evidence that factors such as organizational support, adequate knowledge, and resilience protects the nursers against adverse mental health conditions and support the staff’s psychological wellbeing.

In the highly demanding work environment dealing with common goodness like people’s health (Abid and Contreras, 2022), an empowering and supportive leadership behavior toward the employees is crucial for enhancing staff’s wellbeing. This leadership is called sustainable leadership. Sustainable leadership aims to strike a balance between an organization’s human resources, profitability, and the planet over its lifecycle (McCann and Holt, 2010). According to Choi (2021), sustainable leadership behavior is an umbrella framework involving servant, authentic and ethical leadership characteristics that have in common an empowering and supportive behavior toward the employees. Sustainable leadership is highly effective in environmental challenges because it emphasizes environmental diversity, sustained learning, efficient stakeholder management, development of resources, long-lasting success, amicable relationships with the workforce, and social, ethical, and responsible behavior. In light of the paucity of empirical studies about the significance of sustainable leadership and its relation to wellbeing, there is little research in healthcare settings and environmental considerations confronting Asia. Thus, framed in the AMO theory of Appelbaum et al. (2000), who consider the opportunity for participation as a core element, we propose that sustainable leadership exerts influence on employees’ wellbeing. The AMO theory involves two other components: ability and motivation, which will be addressed with the other variables included in the proposed model.

In addition to sustainable leadership support and encouragement to participate, doing the right things in the right manner in a professional team in highly stressful times is linked to knowledge sharing, e.g., sharing procedural knowledge (Akgün et al., 2008). In the knowledge management field, procedural knowledge involves sequential actions, procedures, and steps to solve problems through the application of automated techniques (Aydın and Özgeldi, 2019; Wuryaningrum et al., 2020). For example, effective procedures that help to decrease the virus infection risk should be shared among the healthcare staff (Petrella et al., 2021) to reduce the anxiety generated by the unknown. Thus, in pandemic times and the usual complex conditions of healthcare settings, we consider that procedural knowledge is a relevant factor in promoting the wellbeing of healthcare staff. From the AMO framework, the ability is the component related to how people possess the required knowledge and skills to perform well, reducing the anxiety produced by feeling overcome by performing their duties properly. Under this framework, procedural knowledge sharing enhances the ability of the health staff (De Kock et al., 2021), which could influence the employees’ wellbeing. We argue that in this highly demanding work environment with changing policies, procedural knowledge sharing has a significant impact on the wellbeing of health care staff, and sustainable leadership could trigger procedural knowledge sharing.

The last component of the AMO theory is the motivation to serve others, an individual factor that in the healthcare profession is considered compassion. Compassion as an individual factor for caring about others is crucial for the health care staff’s wellbeing. Lilius et al. (2011, p. 874) defined “compassion as the reliable capacity of members of a collective to notice, feel and respond to suffering.” Compassion is a prosocial, positive emotion that involves feeling for and wanting to help others in distress (Goetz et al., 2010). At the organizational level, compassion flows when individual interests are aligned with the organizational value system (Renzenbrink, 2011).

In a nutshell, for our explorative study, the mentioned factors are of interest because they could impact the wellbeing of healthcare staff in times not only of normal high pressure but in a health crisis that leads to frequently changing policies because of unexpected situations such as the pandemic (Digby et al., 2021). Following this argumentation, we propose a research model where sustainable leadership influences employees’ wellbeing in healthcare settings and that such a relationship could be mediated by procedural knowledge and compassion. Based on the above arguments, the research questions are:

RQ1: Could sustainable leadership influence the wellbeing of the healthcare staff?

RQ2: How are procedural knowledge and compassion at work associated with employees’ wellbeing in healthcare settings?

## 2. Literature review and hypotheses

### 2.1. Sustainable leadership and wellbeing

Sustainable leadership is a new domain of effective leadership, which has been established recently to cope with issues related to sustainable development (Iqbal and Ahmad, 2021). Long-term perspectives, systemic innovation, workforce development, and quality are the foundations of sustainable leadership practices. To illustrate how sustainable leadership is still operationalized, we summarized different studies: On one hand, Avery and Bergsteiner (2011) defined a broad scope of sustainable leadership practices by including the corporate social responsibility (CSR) concept from a strategic management perspective. Further, Lee (2017) integrate internal CSR and sustainable human resource management (HRM) elements into sustainable leadership with diversity management, employee development, organizational justice, progress development and work-life balance impact satisfaction, motivation, and performance. On the other hand, Choi (2021) operationalized sustainable leadership as concrete behavioral practices related to servant, authentic and ethical leadership styles.

Moreover, Hallinger and Suriyankietkaew (2018, p. 3) considered the foundation for sustainable leadership in “Rhineland approach capitalism in Germany” focusing on social care, highlighting the responsibility for employees and society. Based on their review, the following features are summarized in a conceptual framework: sustainable leadership links the long-term vision and organizational goals to the society’s welfare, ethical behavior, social responsibility of leaders and the organization, stakeholder engagement to such vision, and innovation capacity for an open system. Sustainable leadership and its associated values, combined with knowledge and experience, increase the output of the CSR’s triple bottom line performance, that is, social, ecological, and economic performance (van Veldhoven and Peccei, 2015; Hallinger and Suriyankietkaew, 2018).

There is some evidence regarding the impact of sustainable leadership on sustainable performance (Iqbal et al., 2020a), and employee satisfaction (mainly influenced by valuing employees, ethical behavior, and shared vision; Suriyankietkaew and Avery, 2014). Individualized consideration “serves as a carrot” to satisfy employee’s personal needs. Recently, Choi (2021) showed that managers’ sustainable leadership significantly impacts employee wellbeing, especially when it is oriented to servant and authentic leadership practices. Similarly, virtuous leadership behavior, which is linked to sustainable leadership by its ethical approach, has shown its influence on work-related wellbeing (affect, job satisfaction, and work engagement), whereas trust in the leader served as a mediator (Hendriks et al., 2020). Sustainable leadership strives to improve the lives of all stakeholders while generating profits for the now and future. It emphasizes the fundamental value of sustainability at the personal, corporate, and societal levels. Supported by the above, we formulate the following hypothesis:

*Hypothesis 1*: Sustainable leadership is positively associated with wellbeing.

### 2.2. Sustainable leadership and procedural knowledge

Workplaces are learning environments that can provide their employees with opportunities through a proper condition, for learning in everyday work (Fuller and Unwin, 2004); among these conditions are autonomy, knowledge sharing, managerial support, competence and career among others are learning conditions that support the management of stressful work (Gustavsson and Lundqvist, 2021). Organizational knowledge can be declarative or procedural. While declarative knowledge is based on facts, propositions and events, procedural knowledge refers to specific knowledge about how things are done (Kyriakopoulos, 2011), which is important in the high demanding work environment of healthcare. Procedural knowledge is an organizational knowledge refers to specific knowledge about how things are done (Kyriakopoulos, 2011), involves a set of unit procedures organized for solving a specific purpose (Song et al., 2011). Managerial support and knowledge sharing in the workplace are good contextual conditions that encourage the learning process and are important for managing stressful work conditions (Gustavsson and Lundqvist, 2021). Kim and Park (2020) revealed a significant impact of transformational leadership as well as organizational climate on knowledge sharing, further a mediation path of leadership through knowledge sharing on organizational learning. Further Le and Lei (2019) confirmed the path of transformational leadership on knowledge sharing moderated by high perceived organizational support, further knowledge sharing mediates the impact of leadership on product and process innovation. This evidence gives support for the impact of the immediate work context factors on work outcomes. Effective leadership behavior is a significant important impact factor for knowledge management.

But how does sustainable leadership influences procedural knowledge sharing? Tuan (2016) provided evidence that servant leadership (as part of sustainable leadership, see Choi, 2021) enhanced knowledge sharing in a public organization moderated by public service motivation and CSR. Lee et al. (2016) found a significant impact of top management support and clan culture on knowledge sharing, which serves as a mediator in process improvement success. Khalil et al. (2021) investigated the impact of sustainable leadership on knowledge sharing. Four dimensions of sustainable leadership (i.e., sustainability leadership, ethical leadership, mindful leadership, and servant leadership, in line with Choi, 2021) impact knowledge sharing. Moreover, Chaman et al. (2021) evidenced the impact of ethical, transformational and passive avoidant leadership on knowledge sharing mediated by introjected motivation as ethical leadership is an element of sustainable leadership. Sustainable leaders motivate and inspire staff to share new ideas and stimulate creativity, resulting in the organization’s constant improvement. Such methods also ensure that employees will embrace new techniques for doing business. Sustainable leaders encourage knowledge exchange throughout firms to boost employees’ ability to think outside the box.

According to Hart’s (1995) natural resource-based view (NRBV) perspective, environmentally friendly resources are required to improve organizational performance and provide a sustainable competitive advantage. This study utilizes sustainable leadership as a resource in order to be ecologically friendly. Sustainable leaders identify sustainability issues, communicate long-term visions, establish polices for green management, and promote green activities (Avery and Bergsteiner, 2011). While maintaining strong relationships with many stakeholders, sustainable leaders scan and monitor potential external environment changes. Additionally, it enhances organizational performance by minimizing operating expenses and identifying possible business opportunities. Therefore, we conclude that sustainable leadership behavior and sharing of procedural knowledge are significant organizational/immediate work context variables for employee wellbeing in the health care profession. As Khalil et al. (2021) and Chaman et al. (2021) showed, sustainable leadership behavior should enhance procedural knowledge in a highly stressful work environment in health care. The current study postulates that an organization might use sustainable leadership as a resource to develop procedural knowledge. Thus, we postulate the following hypothesis:

*Hypothesis 2*: Sustainable leadership is positively associated with procedural knowledge.

### 2.3. Procedural knowledge and compassion

Procedural knowledge refers to specific knowledge about how things are done (Kyriakopoulos, 2011). In the organizations, procedural knowledge is crucial to understand concepts and develop the strategy to find problem solutions (Richter-Beuschel et al., 2018; Ismail, 2020). This knowledge is more tacit; people are hardly aware of it and is acquired from experience, which makes it difficult to be transferred (Gupta and Govindarajan, 2000) and measured (Richter-Beuschel et al., 2018). The effectiveness of procedural knowledge requires an organizational environment that allows access to knowledge and promotes collaborative practices that encourage collective knowledge that can be inserted into organizational routines (Nieves et al., 2016).

The workplace learning perspective is supported by situated learning which assumes that learning goes beyond an individual process, and includes learning conditions and learning environment (Evans et al., 2006). Long-term superior performance could be guided by organizational learning attitudes, behaviors, and techniques. Natural resource-based view (NRBV) theory states that businesses can generate dynamic capability by establishing, reconfiguring, and integrating their capabilities to thrive in a dynamic market if they use resources as a foundation for sustainable competitive advantages. Organizational learning is seen as a dynamic capacity since it helps organizations to adjust continuously to market demands. Dynamic capability is based on how knowledge sources are created, collected, integrated, shared, and used. Organizational learning in the context of knowledge-based dynamic capacities entails the generation of new knowledge and the incorporation of new pieces of explicit knowledge into institutional memory. Dynamic capability drives greater performance since the learning organization encourages the generation of knowledge and its application activities.

Compassioned people create an environment of acceptance and harmony at work due to the recognition that the human experience is not perfect. They accept and recognize that everybody makes mistakes, which enables them to be more connected with the individual difficulties of others (Neff and Costigan, 2014). As a virtuous circle, a compassionate environment where it is recognized that the job can be improved and some mistakes are accepted fosters the employees to continuously strengthen their learning, improving performance in the procedures that their work implies.

Therefore, we propose that procedural knowledge effectiveness could strengthen the relationships between employees, colleagues and supervisors, contributing to the creation of a culture of compassion, which is characterized by helping behaviors (Piff et al., 2010), generosity (Saslow et al., 2013) and forgiveness (Worthington and Scherer, 2004). Supported by the above contention, we posit the following hypothesis:

*Hypothesis 3*: Procedural knowledge is positively associated with compassion.

### 2.4. Compassion and wellbeing

Compassion at work contributes to building high-quality relationships, enhancing relational resources such as loyalty, trust, and the connectedness between people, which leading healing people in struggling situations (Dutton et al., 2007). According to Ryan and Deci (2001), there are two main philosophical viewpoints on well-being: one is happiness-oriented (i.e., hedonism), which defines well-being as the subjective experience of happiness; the other is eudaimonism, which focuses on realizing human potential and sees well-being as the result of personal success, self-actualization, or self-positioning. Compassion can be understood as an indicator of intrapersonal wellbeing, as a way of relating to oneself and others and promoting eudaimonic happiness (Neff and Costigan, 2014). From the eudaimonic approach, wellbeing and happiness are considered subjective experiences that tend to be stable over time and involve life satisfaction and positive affect (Howell et al., 2007).

There is evidence about the effect of compassion on nurses’ wellbeing. In this regard, Wahl et al. (2018) found that compassion allows nurses to feel a sense of joy, satisfaction, and fulfillment in their professional work, connecting with their patients and their suffering, allowing the nurses to fulfill their professional and/or personal commitment to finding meaning in their work. Consequently, compassion increases employee commitment and decreases turnover and absenteeism (Dutton et al., 2007), all those personal and contextual work characteristics are related to employees’ wellbeing.

According to Bag et al. (2022), higher levels of self-compassion are associated with better wellbeing. Muris et al. (2018) also confirm that self-compassion strongly predicts students’ mental health. Additionally, Zessin et al. (2015)’s meta-analysis revealed a substantial positive relationship between self-compassion and wellbeing in general adult samples. Therefore, we propose that compassion can be related to wellbeing and posit the following hypothesis:

*Hypothesis 4*: Compassion is positively associated with wellbeing.

### 2.5. Procedural knowledge and compassion as mediators

This research aims to explore the mediator role of procedural knowledge and compassion in the relationship between sustainable leadership and wellbeing. Sustainable leaders promote a psychologically safe working environment that promotes effective learning within an organization (Iqbal et al., 2020b) through collaborative practices that influence procedural knowledge (Nieves et al., 2016). In turn, procedural knowledge could encourage the employees wellbeing due to this kind of knowledge supporting people in accomplishing their work and handling difficulties related to it in their everyday work (Kimmerle et al., 2010).

Bakker and Demerouti (2017) assert that learning is facilitated by working conditions and provides employees with resources to handle high work demands. Workplaces that enable learning conditions provide employees with ample resources for managing stressful work (Gustavsson and Lundqvist, 2021), which will contribute to the employees’ wellbeing. Therefore, workplace learning is a significant way to reduce stress and improve the employees’ health (Holman and Wall, 2002; Panari et al., 2010) by enhancing the employees’ ability to cope with stressful situations and high work demands (Proost et al., 2012). Thus, a workplace environment that provides learning conditions helps employees to manage stressful work by relieving the imbalance between work demands and resources (Gustavsson and Lundqvist, 2021). In addition, a compassionate work environment where there is access to learning opportunities is a crucial resource for dealing with demanding work by reducing stress (Gustavsson and Lundqvist, 2021). Such a work environment can emerge in compassionate organizations. Compassionate behaviors are learned in the organizations according to how employees interact with each other (Banker and Bhal, 2020), knowledge is shared, and employees experience open communication between them (Gustavsson and Lundqvist, 2021).

According to Banker and Bhal (2020), managers influence the creation of compassionate organizations, and sustainable leadership seems to accomplish the required qualities to it. These leaders have a long-term vision, broader goals that benefit society, ethical behavior, and social responsibility (Hallinger and Suriyankietkaew, 2018). On the contrary, leaders that are excessively focused on short-term goals, exert high pressure on employees, and are not trustable promote organizations with low levels of compassion. On the other hand, sustainable leadership influences organizational learning because of its long terms objectives (Sharma and Lenka, 2019) and the knowledge-sharing culture (Kantabutra and Avery, 2013). Sustainable leadership promotes a vision supported by organizational values, including moderation, mutual respect, and the value of individuals; these values underlie the employees’ satisfaction, commitment, and performance (Hargreaves and Fink, 2007).

Sustainable leaders share a long-term vision and promote knowledge dissemination in the companies, by maintaining open communication (Park and Kim, 2018), framed in ethical behaviors (Kantabutra and Avery, 2013). Ethical practices from the leaders move their followers to become sensitized to peers’ problems and be more compassionate, increasing compassion in their companies, and compassion is a driver for wellbeing (Manrique-de-Lara and Viera-Armas, 2019). Thus, sustainable leaders promote compassionate work environments through the values such as integrity, empathy, accountability, authenticity, presence, dignity (Shuck et al., 2019), empathy, ethical/moral values, and supportive organizational culture joined to favorable human resource practices (Banker and Bhal, 2020), all of which will result in the wellbeing of employees. Empathetic leaders as sustainable leaders are needed to build compassionate organizations where there is a shared moral virtue that strengthens the social and emotional relationships between employees and between them and their organizations, making virtuous organizations (Karakas et al., 2017).

Addressing compassion in healthcare organizations is especially relevant because care without compassion might prove dangerous to patients (Renzenbrink, 2011) and even unethical (Austin et al., 2009). Unfortunately, not all hospitals run with compassion, and there is insufficient knowledge to explain why despite its relevance. In this regard, Banker and Bhal (2020) stated the necessity to identify factors that promote compassion in these organizations.

In conclusion, current study envisages that how procedural knowledge relates to compassion of health care workers in social context drawing on AMO framework (Appelbaum et al., 2000). According to AMO theory, three independent work systems which shape how an employee behave in an organizational setting are ability, motivation and opportunity accorded by employers. Sustainable leaders focus on knowledge-sharing culture and organizational learning (Kantabutra and Avery, 2013; Al-Zawahreh et al., 2019). We built arguments based on AMO theory that sustainable leaders strengthen their followers with the ability, opportunity and motivation to enhance their wellbeing by the mediating role of procedural knowledge and compassion. Following the previous arguments, we propose that the relationship between sustainable leadership and employees’ wellbeing may be mediated by procedural knowledge and compassion, proposing the following hypotheses:

*Hypothesis 5*: Procedural knowledge mediates the relationship between Sustainable leadership and wellbeing.

*Hypothesis 6*: Compassion mediates the relationship between sustainable leadership and wellbeing.

*Hypothesis 7*: Procedural knowledge and compassion sequentially mediate the relationship between sustainable leadership and wellbeing.

**Figure 1:**
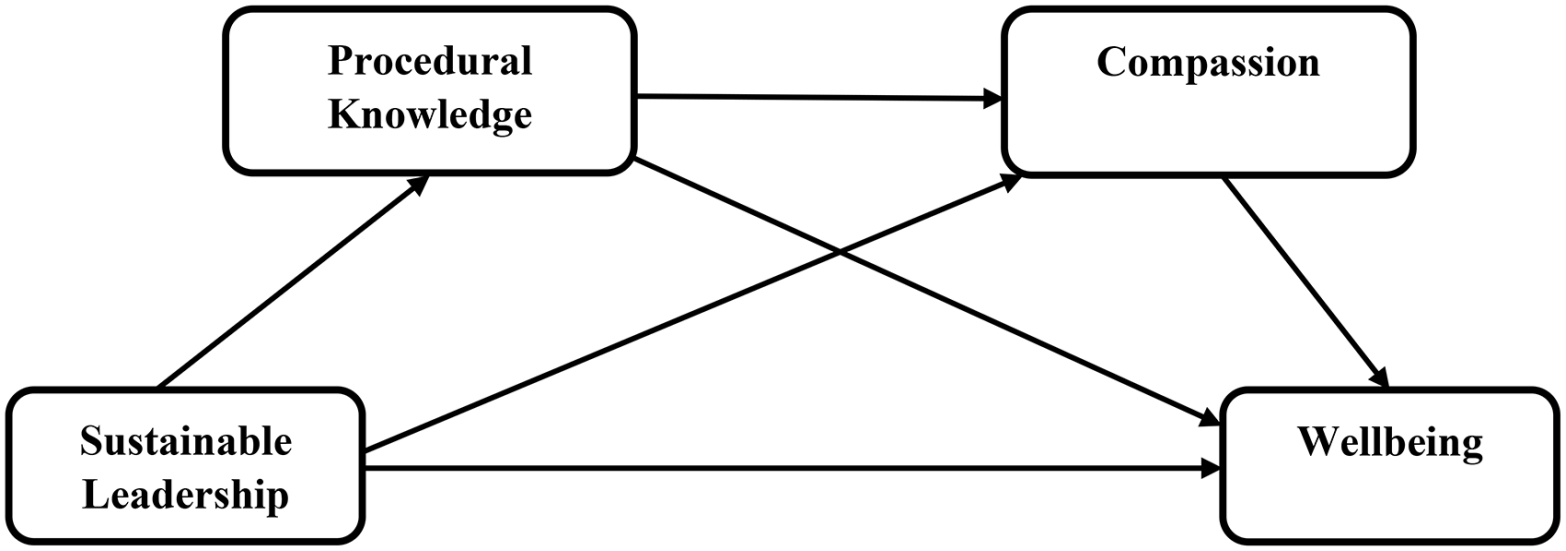
Research model.

In summary, all direct and indirect paths are visualized in Figure 1.

## 3. Materials and methods

### 3.1. Participants and procedure

Pakistani physicians and nurses employed in public and private hospitals are the focus of the current study. Questionnaires were distributed to doctors and nurses in private and public hospitals in Lahore, Faisalabad, and Islamabad. We have gathered data from these cities because these cities (i.e., Lahore, Faisalabad and Islamabad) are deemed to be major cities in the province of Punjab (Baig et al., 2006; Ghafoor et al., 2021; Majeed et al., 2022). The health sector is believed to have a crucial role in the industrial progress of any economy (Doeksen et al., 1997) and its social impact on the lives of individuals (Sen and Östlin, 2008). We concentrated on a particular industry because “unknown sources of variation attributable to organization type could be controlled” (Near et al., 2004). In addition, recent research has collected samples from the health sector during pandemics (Sethi et al., 2020). The data was collected during first wave of coronavirus pandemic so as to let the data reflect the true picture of the hospital works setting in the data of current study.

The same argument applies to our selection of the health industry as a study sample. Due to the unpredictability of data collection from frontline healthcare personnel during the coronavirus pandemic, a split questionnaire survey design (SQSD) is employed to collect study data. Prior research provides a foundation for employing the split-questionnaire technique to lessen respondents’ answer burden (Raghunathan and Grizzle, 1995; Ahmed et al., 2015; Farooqi et al., 2017). Using SQSD, the questionnaire is divided into two portions, A and B, and sent to two distinct groups of respondents. Demographic questions were requested from both groups. The current study complies with the data collection recommendations of Podsakoff et al. (2003), which state that independent and dependent variable data should be collected at distinct times to prevent common method bias. The current study employed a two-waved time-lagged study design to reduce the source bias. At time 1 (T1), data were collected for sustainable leadership, procedural knowledge and wellbeing measures. Almost after an interval of 1 month, i.e., at time 2 (T2), data were collected for the measure of compassion from the same respondents.

In addition, respondent anonymity is ensured from the earliest phases of data collection to reduce social desirability bias (Nederhof, 1985). According to Guadagnoli and Velicer (1988), a sample size of 300 to 400 is an excellent representation of the population. Consequently, using the split questionnaire survey methodology, we presented the questionnaire to 450 participants *via* online (Google form) and printed forms. Set A of the questionnaire was administered to 225 respondents, while Set B was administered to 225 respondents. After collecting replies in hard and soft form from both sets of respondents and removing missing responses at either time (i.e., T1, T2) and outliers, we merged both datasets to get 366 total responses.

The frequency distribution of demographic responses showed that 135 male respondents represented 36.9% of the sample, while 231 female respondents represented 63.1% of the sample size. Participants were grouped into five age groups ranging from under 25 to over 55. Age distribution study reveals that most respondents were between the ages of 25 and 34. In addition, a frequency distribution of hospital categories reveals that out of 366 respondents, 248 (67.8%) belonged to public sector hospitals and 118 (32.2%) to private sector hospitals. Furthermore, frequency analysis for marital status indicates that the majority of respondents, 223 (60.9 percent) out of 366, were married.

### 3.2. Sample size calculation

A preliminary power analysis was conducted to determine the ideal sample size. Power analyses were performed by using G*Power (G*Power, 2020) version 3.1.9.7 (Institut fur Experimentelle Psychologie, Heinrich Heine Universitat, Dusseldorf, Germany) and input parameters (effect size f2 = 0.377) for the sample size computation were based on the squared multiple correlation p2, yielding a minimum of 34 participants. Furthermore, we also performed *a priori* sample size calculation for sample power even with a very small, i.e., 0.04 effect size, 0.05 α error probability, and 80% sample power, yielding a minimum of 277 participants. The large sample size makes the findings more valid and generalizable, so we targeted 450 participants. In the *post hoc* analysis, the sample of 366 participants with a small effect size f2 = 0.05 provided a power of 0.96, which is statistically enough to make conclusions.

### 3.3. Measures

*Sustainable Leadership* was measured by adopting a 4-item scale developed by Di Fabio and Peiró (2018). Cronbach’s alpha = 0.93. *Procedural knowledge* was measured by adopting a 4-item scale developed by Akgün et al. (2008). Cronbach’s alpha = 0.94. *Compassion* was measured using a 3-item scale adapted by Lilius et al. (2008). Cronbach’s alpha = 0.75. *Wellbeing* was measured using a 5-item scale adopted by Han (2020). Cronbach’s alpha = 0.91. A five-point Likert type scale (1 = strongly disagree to 5 = strongly agree) was used for all the measures except for wellbeing, which was measured on a seven-point Likert type scale (1 = strongly disagree to 7 = strongly agree).

### 3.4. Analytical strategy

To conduct data analysis and test the stated hypotheses, we adhered to the methods used by previous researchers. Specifically, the confirmatory factor analysis (CFA) was conducted using version 24 of the AMOS (IBM, Armonk, United States, 2014; maximum likelihood) program to assess the factorial structure and suitability of our proposed four-factor measurement model. Following the CFA, hypotheses were evaluated with the PROCESS macro analysis. The PROCESS macro (Hayes, 2012) analysis was chosen because, according to bootstrap sampling, it has been acknowledged as a reliable and rigorous method for assessing the magnitude of conditional indirect effects (Abid et al., 2020).

## 4. Results

### 4.1. Confirmatory factor analysis

Before testing the hypotheses, a measurement model was tested with the help of CFA using AMOS 21.0 to ensure the goodness of fit for the variables under study. To assess the fit indices for CFA, this study used Chi-square test statistic (χ2/df), GFI (goodness of fit), AGFI (adjusted goodness of fit index), TLI (Tucker-Lewis Index), CFI (comparative fit index), and RMSEA (root mean square error of approximation). The values of Chi-square test statistic < 3, GFI, AGFI, TLI, CFI scores > 0.90, and RMSEA scores < 0.08 signify an acceptable fit (Kline, 2015). The measurement model comprised of four factors: sustainable leadership, procedural knowledge, compassion, and wellbeing showed a good fit as per CFA. According to our expectations, the our four-factor model representing fit the data well (χ2/df = 2.54, GFI = 0.948, AGFI = 0.916, TLI = 0.963, CFI = 0.973, and RMSEA = 0.065). Furthermore, our four-factor measurement model is considerably better than the alternate two and one-factor models (see Table 1). These results revealed that examining the four variables as separate constructs is justified.

**Table 1:** Fit indices of measurement and alternative models.

Models	χ2/df	GFI	AGFI	TLI	CFI	RMSEA
Four-factor model	2.54	0.948	0.916	0.963	0.973	0.065
Three-factor model	24.43	0.706	0.551	0.442	0.569	0.253
Two-factor model	27.90	0.651	0.487	0.359	0.486	0.271
One-factor model	32.81	0.565	0.372	0.243	0.380	0.295

Three-factor model: sustainable leadership and procedural knowledge were combined; Two-factor model: sustainable leadership and procedural knowledge were combined and compassion and wellbeing were combined; GFI, goodness of fit index; AGFI, adjusted goodness of fit index; TLI, Tucker-Lewis coefficient; CFI, comparative fit index; RMSEA, root mean square error of approximation.

### 4.2. Convergent and discriminant validity

Convergent validity is the measure to which a statistic significantly corresponds to other alternative measures of the same constructs (Hair et al., 2017). To demonstrate the convergent validity, average variance extracted (AVE) and composite reliability are examined (Fornell and Larcker, 1981). The value of AVE should be >0.50 and composite reliability should be >0.70. All the study constructs passed the minimum AVE and composite reliability criteria, so convergent validity is achieved. Divergent validity is another name for discriminant validity; it refers to the degree to which one construct differs from the others. It is determined using the square root of the AVE. The square root of the AVE of the construct should be larger than its correlations with other variables. The result indicates that the square root of the AVE of the selected constructs is greater than the correlations of constructs (Fornell and Larcker, 1981) (see Table 2).

**Table 2:** Convergent and divergent validity.

Variables	CR	AVE	MSV	ASV	1	2	3	4
1. Procedural knowledge	0.93	0.82	0.13	0.05	**0.91**			
2. Sustainable leadership	0.92	0.80	0.08	0.04	0.14	**0.89**		
3. Compassion	0.77	0.53	0.13	0.09	0.36	0.28	**0.73**	
4. Wellbeing	0.76	0.51	0.08	0.03	0.13	0.10	0.28	**0.71**

SL, sustainable leadership; PK, procedural knowledge; comp, compassion; WB, wellbeing; CR, composite reliability; AVE, average variance extracted; MSV, maximum shared variance; ASV, average squared shared variance. The bold values means square root of average variance extracted.

The descriptive statistical analysis and correlations of variables are presented in Table 3. The correlation coefficients fall in the expected direction and provide early evidence for our study’s findings. Positive and significant relationships exist between sustainable leadership and procedural knowledge (r = 0.23, *p* < 0.01) and sustainable leadership and wellbeing (r = 0.19, *p* < 0.01). There was a positive correlation between procedural knowledge and compassion (r = 0.41, *p* < 0.01) and wellbeing (r = 0.21, *p* < 0.01). Moreover, there was a strong correlation between compassion and wellbeing (r = 0.22, *p* < 0.01). Initial support for the postulated relations was presented by these significant correlations in the expected direction.

**Table 3:** Mean, standard deviation, and correlations.

Variable	Mean	SD	1	2	3	4	5	6	7	8
1. Gender	−	0.48	1							
2. Age	−	0.99	0.12^*^	1						
3. Marital Status	−	0.53	−0.06	0.32^**^	1					
4. Hospital Type	−	0.47	−0.08	−0.54^**^	−0.19^**^	1				
5. SL	3.63	0.73	0.03	−0.11^*^	−0.04	0.08	1			
6. PK	3.85	0.74	0.07	−0.01	−0.14^**^	0.06	0.23^**^	1		
7. COMP	3.69	0.63	0.12	−0.02	−0.06	0.01	0.34^**^	0.41^**^	1	
8. WB	4.91	1.45	0.03	−0.29^**^	−0.08	0.10	0.19^**^	0.21^**^	0.22^**^	1

*N* = 366. SL, sustainable leadership; PK, procedural knowledge; COMP, compassion; WB, wellbeing.

** Correlation is significant at the 0.01 level (2-tailed).

* Correlation is significant at the 0.05 level (2-tailed).

**Figure 2:**
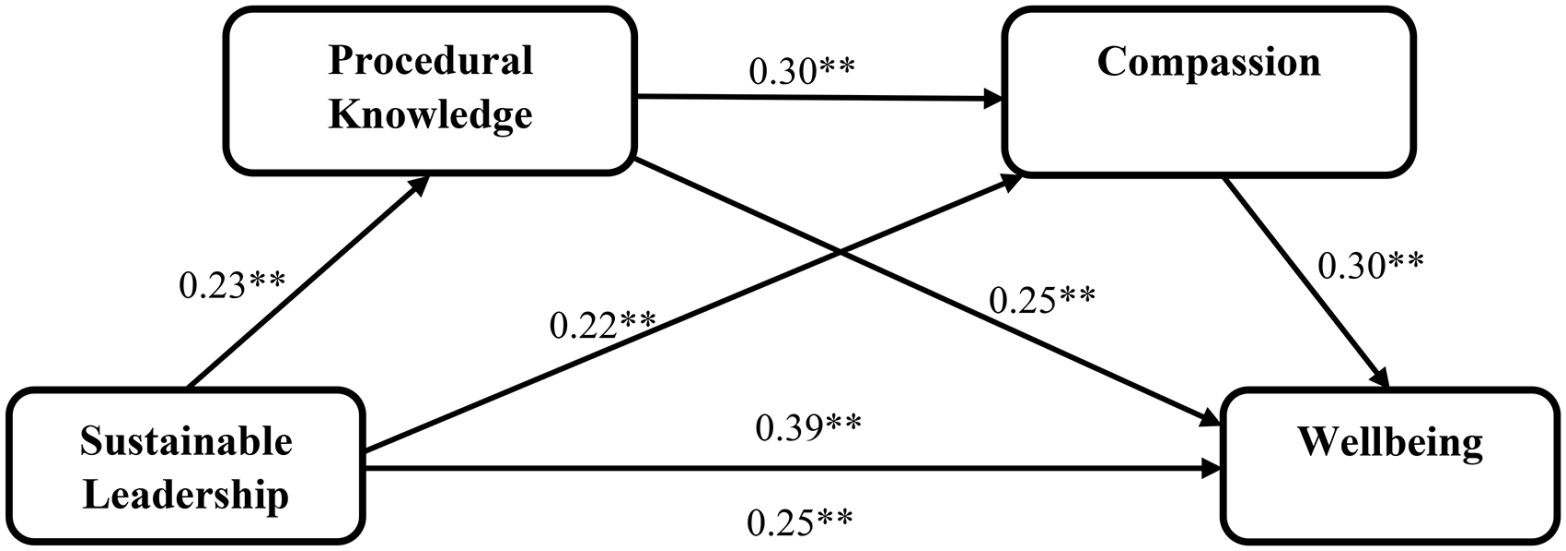
Sequential mediation model linking sustainable leadership and wellbeing.

To test our sequential mediated model and all direct and indirect hypotheses further, we used Hayes’ process (Hayes, 2012), which according to Field (2013) is “by far the best way to tackle sequential mediation.” According to our hypothesized model, procedural knowledge and compassion sequentially mediate the relationship between sustainable leadership and wellbeing. Therefore, we used a Hayes process model 6 to test our theory on a sample of 366 with parameter estimates based on 2,000 bootstrap samples. The bias-corrected and accelerated 90% confidence intervals were then examined. The results of the PROCESS analysis show that sustainable leadership significantly predict wellbeing β = 0.25, 90% CI [0.068, 0.422], t = 2.28, *p* = 0.02 and procedural knowledge β = 0.23, 90% CI [0.144, 0.315], t = 4.42, *p* = 0.00, hence supporting hypothesis 1 and 2, respectively (see Table 4). Furthermore, the results show the procedural knowledge significantly promote compassion among employees β = 0.30, 90% CI [0.231, 0.365], t = 7.34, *p* = 0.04, hence supporting hypothesis 3. The findings also show that compassion significantly and positively predicts wellbeing of the employees β = 0.29, 90% CI [0.077, 0.511], t = 2.23, *p* = 0.03, hence supporting hypothesis 4. The results of hypothesis testing are depicted in Figure 2.

**Table 4:** Results of sequential mediation (direct and indirect effects).

Model pathways	Β	SE	t	p	LLCI	ULCI
**Direct Effect**						
H1: SL → WB	0.25	0.11	2.28	0.02	0.068	0.422
H2: SL → PK	0.23	0.05	4.42	0.00	0.144	0.315
H3: PK → COMP	0.30	0.04	7.34	0.00	0.231	0.365
H4: COMP → WB	0.29	0.13	2.23	0.03	0.077	0.511
	Estimated	BootSE		BLLCI		BULCI
**Indirect effect**						
H5: SL → PK → WB	0.028	0.015		0.006		0.056
H6: SL → COMP → WB	0.033	0.016		0.009		0.063
H7: SL → PK → COMP → WB	0.010	0.006		0.002		0.020

BootSE, Bootstrapped standard error estimate; BLLCI, Bootstrapped lower limit confidence interval; BULCI, Bootstrapped upper limit confidence interval; 90% bootstrap, 2,000.

In addition to the direct paths, we find the significant indirect effect of procedural knowledge in the relationship between sustainable leadership and wellbeing (Effect = 0.028, 90% CI [0.006, 0.056]), hence supporting hypothesis 5. Moreover, results indicated the significant indirect effect of compassion in the relationship between sustainable leadership and wellbeing (Effect = 0.033, 90% CI [0.009, 0.063], hence supporting hypothesis 6). Finally, the results of sequential mediation of procedural knowledge and compassion in the relationship between sustainable leadership and wellbeing are also significant and positive (Effect = 0.010, 90% CI [0.002, 0.020], hence supporting hypothesis 7).

## 5. Discussion

Organizations have grown more concerned with their employees’ wellbeing in recent years as it can benefit them (Bakker and Oerlemans, 2011; Taheri et al., 2019; Busch et al., 2021). The current study examined the relationship between sustainable leadership, procedural knowledge, compassion and wellbeing of employees using the AMO framework (Appelbaum et al., 2000). The results of the current study supported our hypotheses: sustainable leadership is indirectly related to employee wellbeing *via* the sequential mediation of procedural knowledge and compassion. Study findings suggest that sustainable leaders can trigger procedural knowledge in a stressful work environment in healthcare institutions. Further, the role of a sustainable leader in facilitating a compassionate environment to beget compassion among employees leads to their wellbeing. These findings fill in gaps in the research on employee wellbeing while adding to earlier studies.

As a matter of fact, wellbeing is considered a vital component of positive organizational psychology (Bakker, 2015; Martín-del-Río et al., 2021) and is critical to the mental health of individuals (Coverdale and Long, 2015; Wilkes et al., 2019). From the contextual point of view, our study findings suggest that the effect of sustainable leadership on wellbeing is passed through enhancing the individuals’ dynamics in the wake of crises like coronavirus pandemic, i.e., *via* sequential mediation of procedural knowledge and compassion. In line with AMO framework (Appelbaum et al., 2000; Kellner et al., 2019), the ability of healthcare workers is enhanced as a result of procedural knowledge accorded by sustainable leaders and as a result of which, the healthcare staff is motivated enough to develop the feeling of compassion among themselves, which leads to wellbeing among them. This outcome is consistent with the paradoxical view of happiness, which is understood as subjective wellbeing by literature (Martin, 2008). Further, our study findings suggest practical implications for fostering subjective wellbeing through individual dynamics.

### 5.1. Theoretical implications

The results of the current study contributed to the literature in several ways. First, previous studies have reported the predictive role of several leadership types on employee wellbeing like, ethical leadership (Kaffashpoor and Sadeghian, 2020; Sarwar et al., 2020), transformational leadership (Tafvelin et al., 2011; Hannah et al., 2020), authentic leadership (Cassar and Buttigieg, 2013; Maher et al., 2017), and servant leadership (Panaccio et al., 2015). Furthermore, no studies have yet examined the role of sustainable leadership in fostering wellbeing among employees, especially in the healthcare context. Therefore, our first contribution is to fill this gap in the literature. Consistent with the AMO framework, the study findings demonstrated that sustainable leadership tends to enhance the ability, i.e., procedural knowledge and motivation and opportunity, i.e., compassion among healthcare workers. Sustainable leadership practices are precursors of employees’ wellbeing. These findings are consistent with prior studies (Hendriks et al., 2020; Choi, 2021), asserting that contextual settings established by leadership are an excellent predictor of wellbeing.

Second, our study extends the understanding of psychological mechanisms built in previous studies (Choi, 2021; Lee and Rhee, 2021) by which sustainable leadership, especially in hospital settings, is able to bring about subjective wellbeing among healthcare workers in times of crisis situations like coronavirus pandemic. Sustainable leadership can act as a stimulus for building procedural knowledge, which can play a crucial role in developing the feeling of compassion among healthcare workers and enhancing their motivation and opportunities in line with the AMO model. Our integrated model with sequential mediation of procedural knowledge and compassion suggested that the dynamic process of bringing wellbeing among employees is not just directly due to leadership but also creating a learning environment to enhance procedural knowledge and promote employee compassion.

Third, our results provide novel insight into the importance of contextual as well as individual dynamics in predicting wellbeing of employees in highly traumatic situations like the coronavirus pandemic. A recent study by Bialobrzeska et al. (2020) found a link between small acts of kindness and how well people feel in times of stress. In line with the AMO research framework (Appelbaum et al., 2000), we found that procedural knowledge afforded to healthcare workers create an environment of compassion, where everyone gets care from each other, hence posing a substantial contribution to the body of literature.

### 5.2. Practical implications

Our results pose practical implications for hospital administrators, policymakers and healthcare workplaces. First, to enhance the abilities and motivation of healthcare employees and improve their subjective wellbeing, hospitals must urge administrators to adopt sustainable leadership across all supervisory levels. This may require broadening their focus from merely meeting the organizational goals to caring about the wellbeing of workers. Healthcare managers must also encourage a learning, especially during traumatic situations like the coronavirus pandemic. In order to achieve this, they have to enhance the procedural knowledge through training programs about how to prevent the risks associated with the coronavirus pandemic. Similar findings were recently reported by Aharon et al. (2021), who demonstrated that training sessions could help improve nurses’ procedural knowledge. Furthermore, hospital administrators and managers might take surveys on the quality of procedural knowledge among workers to get the know-how about the effectiveness of their training.

Second, higher-level interventions should be conducted by leaders of healthcare institutions, providing more feedback to encourage frontline healthcare workers to enhance their tactical knowledge of how things are dealt with to strengthen their procedural knowledge. In this way, healthcare workers are encouraged to consider their working environment and supervisor relationship to enhance their knowledge about processes and activities. Owing to this perspective, traits of sustainable leadership can be opted for at the administrative level in times of crisis to reap the fruitful implication of the study’s framework.

Third, from the point of view of compassion among healthcare workers, a supportive work climate should be promoted, which incorporates positive interpersonal relationships, consideration for one another, workplace autonomy and a specific focus on the wellbeing of workers. In addition, focused HRM practices should be done to create a learning environment to impart procedural knowledge and foster compassion in the workplace. For example, healthcare providers and hospital administrators can launch compassion training besides regular clinical training to foster a culture of kindness and compassion (Callea et al., 2022). The findings of our study reveal that compassion can have a positive relationship with subjective wellbeing, the policymakers and administrators should incorporate the element of compassion while dealing with employees.

### 5.3. Limitations and future research

Some limitations of the current study provide the scope for future research. First, cross-sectional data used in this research does not allow for establishing the casualty between sustainable leadership, procedural knowledge, compassion and subjective wellbeing fully. Although the primary direction of effect follows the direction shown by other research using the AMO framework, longitudinal analysis of the study variables in the future would yield more meaningful cause-and-effect relationships.

Second, a possible single-method bias might be present when using a self-report questionnaire. We assure anonymity to the participants of the study in order to lessen the bias caused by social desirability. Future research, however, will be able to continue the same contention using a new measuring technique, such as, for instance, a daily diary investigation of what employees perform throughout their shifts.

Furthermore, because the surveys were only given to Pakistani frontline healthcare workers, i.e., doctors and nurses, it is possible that the results cannot be generalized to other nations. A future study might thus concentrate on specific industries while extending to other countries.

Finally, future studies using representative samples may examine the generalizability of these findings in representative groups or use additional leadership styles to learn more about how they affect subjective wellbeing in a comparative manner.

## 6. Conclusion

Sustainable leadership is receiving more attention as it is related to increasing the workers’ subjective wellbeing. The AMO framework served as the foundation for this study, which focused on the significance of sustainable leadership in assisting healthcare staff in successfully achieving subjective wellbeing *via* procedural knowledge and compassion during traumatic times. Further research to improve followers’ wellbeing may concentrate on developing leaders to incorporate the elements of servant, authentic and ethical leadership hence making up sustainable leadership and persuading sustainable leaders to concentrate on providing a learning and compassionate work environment.

## Data availability statement

The raw data supporting the conclusions of this article will be made available by the authors, without undue reservation.

## Author contributions

All authors listed have made a substantial, direct, and intellectual contribution to the work and approved it for publication.

## Conflict of interest

The authors declare that the research was conducted in the absence of any commercial or financial relationships that could be construed as a potential conflict of interest.

## Publisher’s note

All claims expressed in this article are solely those of the authors and do not necessarily represent those of their affiliated organizations, or those of the publisher, the editors and the reviewers. Any product that may be evaluated in this article, or claim that may be made by its manufacturer, is not guaranteed or endorsed by the publisher.
